# Cognitive functioning in socially anxious adults: insights from the NIH Toolbox Cognition Battery

**DOI:** 10.3389/fpsyg.2015.00764

**Published:** 2015-06-08

**Authors:** Sonya V. Troller-Renfree, Tyson V. Barker, Daniel S. Pine, Nathan A. Fox

**Affiliations:** ^1^Department of Human Development and Quantitative Methodology, University of Maryland, College Park, MD, USA; ^2^Emotion and Development Branch, National Institute of Mental Health Intramural Research Program, Bethesda, MD, USA

**Keywords:** social anxiety, fluid cognition, crystallized cognition, NIH Toolbox Cognition Battery, executive function

## Abstract

Theory suggests that individuals with social anxiety manifest unique patterns of cognition with less efficient fluid cognition and unperturbed crystallized cognition; however, empirical support for these ideas remains inconclusive. The heterogeneity of past findings may reflect unreliability in cognitive assessments or the influence of confounding variables. The present study examined the relations among social anxiety and performance on the reliable, newly established NIH Toolbox Cognition Battery. Results indicate that high socially anxious adults performed as well as low anxious participants on all measures of fluid cognition. However, high socially anxious adults demonstrated enhanced crystallized cognitive abilities relative to a low socially anxious comparison group.

## Introduction

Social anxiety disorder, which involves excessive fear of social evaluation, impacts as many as 12% of the US population (Kessler et al., 2005). Unlike other forms of anxiety (e.g., specific phobias; Thorpe and Salkovskis, 1995), social anxiety is distinct, well-defined, and marked with anxious behaviors, distress, and physiological arousal in socially threatening contexts (Schlenker and Leary, 1982; Geen, 1991; Rapee and Heimberg, 1997). Rather than representing a discrete, pathological condition, current models view social anxiety disorder as representing the extremes of continuously-distributed levels of anxious symptoms in social situations. Current models link individual differences on this continuous distribution to individual differences in cognition (Clark and Wells, 1995; Eysenck et al., 2007). Discovery of cognitive sequelae of social anxiety is of great interest for both prevention and treatment of social anxiety disorder. However, current evidence and reports of the cognitive profiles associated with social anxiety are inconsistent, possibly due to unreliability in cognitive assessment batteries or failures to control for important confounding variables (Asmundson et al., 1994; Derakshan and Eysenck, 2009).

A number of theories have posited that social anxiety may be associated with differential cognitive processing in a number of cognitive domains when compared to non-anxious controls (Clark and Wells, 1995; Eysenck et al., 2007; Derakshan and Eysenck, 2009). For example, attentional control theory (Eysenck et al., 2007), suggests that anxiety may impair goal-directed attentional systems and increase reliance on stimulus-driven cognitive systems, therefore, negatively impacting processing efficiency via its effects on inhibitory control of attention and attention shifting. Social anxiety may also be accompanied by perturbations in attention shifting (perseveration), poor memory for details, and differential attentional allocation (Clark and Wells, 1995). However, little research has utilized standardized measures of cognition to measure cognitive profiles associated with social anxiety.

Past experimental work investigating the cognitive sequelae of anxiety has focused on two broad cognitive domains: fluid and crystallized cognition. Fluid cognition requires logical thinking, problem solving, and pattern recognition unrelated to specific content domains (Blair, 2006). Common examples include executive functions, such as inhibitory control, working memory, or attention shifting, as well as processing speed and other aspects of attention. In contrast, crystallized cognition is knowledge acquired through learning, usually related to specific content domains (Horn and Cattell, 1966). Common examples include the ability to pronounce irregularly spelt words, receptive word knowledge, or scholastic skills that require explicit learning within a specific domain.

While the association between social anxiety and fluid cognition has been heavily investigated, results remain mixed. For instance, some studies link high levels of anxiety to enhanced performance on tasks of fluid cognitive skills such as attentional orienting (i.e., Eysenck et al., 2007; Henderson et al., 2014), inhibitory control (White et al., 2011), and working memory (Sorg and Whitney, 1992). In contrast, others find reduced performance on measures of on-task attentional focus and processing speed (Cohen et al., 1996; Eysenck et al., 2007), as well as working memory (Asmundson et al., 1994), inhibitory control (Spence et al., 1956; Eysenck and Graydon, 1989; Eysenck et al., 2007), and attention shifting (Cohen et al., 1996; Derryberry and Reed, 2002; White et al., 2011). Finally, still other reports find no association between anxiety and measures of fluid cognition (Asmundson et al., 1994; Graver and White, 2007; O’Toole and Pedersen, 2011).

Fewer studies examine the relations between social anxiety and crystallized cognition, but findings are similarly inconsistent. Most of the relevant work examines the relations between anxiety and academic performance (Wood, 2006; Puklek Levpušček and Berce, 2012). Some work links high levels of social anxiety to high levels of crystallized cognition. For example, Puklek Levpušček and Berce (2012) found that high school students high on social anxiety scales exhibit better academic performance than students low on these scales. However, other studies found no such associations using IQ-related measures (Cohen et al., 1996; Graver and White, 2007).

The heterogeneity of findings across both fluid and crystallized domains emphasizes the need for more work in this area. There is a particular need for studies that simultaneously assess fluid and crystallized cognition, utilizing a standardized, widely-applicable assessment tool while controlling for potential confounding variables. The NIH Toolbox Cognition Battery version 1.0 (see www.nihtoolbox.org for more information) represents such a standardized, widely-applicable tool. It uses seven tasks in order to assess cognitive processing in six sub-domains including: executive function, episodic memory, language, processing speed, working memory, and attention. In addition, the Toolbox uses scores from each task to compute composite scores for crystallized, fluid, and overall cognition. The individual task and composite scores provided by the Toolbox have been carefully standardized and have been shown to be developmentally robust, with very good test-retest reliability (*r*’s = 0.92–0.96), and strong correlations with other established cognitive measures (Akshoomoff et al., 2013, 2014). Finally, the NIH Toolbox Cognition Battery has been normed and validated on 4,859 participants ranging from 3 to 85 years in a representative sample (Beaumont et al., 2013). Thus, the battery provides a solid tool for addressing inconsistent prior work on the relations between social anxiety and cognition. The present study uses this battery to examine the relations between cognition and social anxiety symptoms in early adulthood, in an effort to establish the potential utility of the NIH Toolbox Cognitive Battery.

An additional strength of the Toolbox is its inclusion of tasks that have been previously used with anxious samples. For instance, White et al. (2011) found that poorer performance amongst a sample of children characterized with the temperament of Behavioral Inhibition on the Dimensional Change Card Sort was predictive of later anxious symptoms. Furthermore, the Flanker Inhibitory Control task (Eriksen and Eriksen, 1974) has been widely used in anxiety research with studies showing that anxiety is related to increased error monitoring (Ladouceur et al., 2006; McDermott et al., 2009). To our knowledge, the Picture Sequence Memory, Pattern Comparison Processing Speed, Picture Vocabulary, Oral Reading Recognition, and List Sorting Working Memory tasks have not been used with high socially anxious individuals. The novel application of these tasks with a socially anxious population allows for the investigation of the relation between anxiety and episodic memory, language, processing speed, and working memory within a standardized and replicable framework.

In line with current models of anxious cognition and prior research, we expected that high socially anxious adults will have a different cognitive profile than their non-anxious peers. We expected that, consistent with attentional control theory (Eysenck et al., 2007), socially anxious adults would show deficits in two fluid cognition domains: inhibition (Flanker) and shifting (Dimensional Change Card Sort). Furthermore, consistent with past findings, we expected that high socially anxious adults would perform similarly or better on measures of crystallized cognition when compared to low anxious individuals.

## Materials and Methods

### Participants

Undergraduate students in introductory psychology courses (*N* = 792) completed the self-report version of the Liebowitz Social Anxiety Scale (LSAS-SR; Liebowitz, 1987; Fresco et al., 2001). The LSAS-SR is a 24-item questionnaire that assesses the degree of social anxiety and avoidance during social interactions and performance situations. Participants rate on a Likert scale from 0 (none) to 3 (severe) how much fear/anxiety is experienced in social situations and from 0 (never) to 3 (usually) how often these situations are avoided. The LSAS-SR, which is composed of separate fear and avoidance subscales, is a widely used tool to measure the severity of social anxiety. The LSAS-SR demonstrates excellent psychometric properties and good discriminant validity in differentiating social anxiety from depression and other forms of anxiety (Heimberg et al., 1999). Subjects were recruited for participation in the present study if they scored approximately ±1 SD on the total fear subscale of the LSAS-SR (*M* = 24.63, SD = 12.80), forming two groups of subjects, either high or low on the scale.

The final sample comprised 29 high socially anxious (14 female) and 26 low socially anxious participants (15 female) with a mean age of 19.02 (SD = 1.16) years. All participants provided informed consent and all tasks and measures were approved by the University of Maryland Institutional Review Board.

### NIH Toolbox Cognition Battery

Each participant completed all mandatory tests included in the NIH Toolbox Cognition Battery. No supplementary Toolbox tasks were administered. All tasks were administered by a trained experimenter according to the standards set forth in the NIH Toolbox Administration Manual. Each participant was tested in a private testing room free of environmental distractions. The Toolbox was completed on a Dell Latitude laptop with a dual monitor setup to ensure the participant was not able to view any administration materials.

**FIGURE 1:**
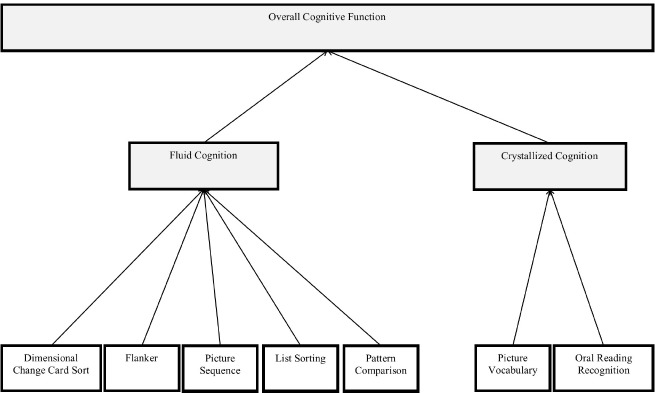
**Structure of NIH Toolbox Cognitive Battery tasks and composites**.

As is standard for all NIH Toolbox assessments, task data was scored and composites were computed using Assessment Center (http://assessmentcenter.net/). The NIH Toolbox Cognition Battery consists of seven tasks: Dimensional Change Card Sort, Flanker Inhibitory Control and Attention, Picture Sequence Memory, Picture Vocabulary, Oral Reading Recognition, Pattern Comparison Processing Speed, and List Sorting Working Memory. In addition to task-specific scores, the NIH Toolbox Cognition Battery provides three composite scores (Figure 1). The Fluid Cognition Composite Score is comprised of the Flanker, Dimensional Change Card Sort, Picture Sequence Memory, List Sorting, and Pattern Comparison tasks. The Crystallized Cognition Composite Score is comprised of the Picture Vocabulary Test and the Oral Reading Recognition Test. The Overall Cognition Composite consists of all tasks included in the Crystallized and Fluid Cognition Composites.

Raw and standardized scores were computed by the NIH Toolbox Assessment Center. For each task and composite, three scores were computed: an unadjusted, age-adjusted, and fully-adjusted score (see Table 1). Fully adjusted scores are computed by comparing the score of the participant to those in the NIH Toolbox nationally representative normative sample to adjust for age, gender, race, ethnicity, and educational attainment (for more information, see the Scoring and Interpretation Guide at www.nihtoolbox.org). To examine cognitive functioning in the most standardized framework, fully adjusted scores were used for all analyses.

**TABLE 1:** **Average performance by task across three NIH Toolbox composites**.

****	**Unadjusted**	**Age-adjusted**	**Fully adjusted**
Overall Cognition Composite	138.94 (11.19)	135.49 (13.85)	133.03 (14.47)
Fluid Cognition Composite	134.80 (10.00)	129.89 (13.40)	126.20 (14.05)
Dimensional Change Card	128.09 (9.71)	112.01 (7.95)	107.36 (8.18)
Sort			
Flanker	135.00 (7.89)	117.91 (3.84)	113.05 (4.09)
Picture Sequence Memory	119.51 (11.69)	113.13 (13.49)	111.90 (14.37)
List Sorting Working Memory	116.03 (10.31)	107.08 (11.45)	103.47 (12.49)
Pattern Comparison	130.57 (14.95)	120.47 (17.02)	117.79 (17.37)
Crystallized Cognition	119.70 (9.20)	115.75 (14.24)	111.76 (14.33)
Composite			
Picture Vocabulary Test	114.59 (8.17)	108.61 (12.83)	107.20 (13.14)
Oral Reading Recognition	122.71 (11.18)	114.89 (12.67)	112.42 (12.38)
Test			

Means and standard deviations in parentheses.

### Participant Inclusion

A total of 55 participants completed of NIH Toolbox Cognitive Battery. Of these participants, six participants were excluded from analysis for participant reporting error (1), experimenter or technical error leading to missing task data (3), and incomplete data (2). The final sample consisted of 26 high socially anxious (14 female) and 23 low socially anxious participants (12 female) with a mean age of 19.02 (SD = 1.16) years (see Table 2).

**TABLE 2:** **Demographics, descriptive statistics, and average performance by group**.

****	**Low social anxiety**	**High social anxiety**
Age	18.87 (1.014)	19.15 (1.29)
Gender (% female)	52.2	53.8
LSAS total score	19.96 (9.97)	76.92 (11.54)
Overall Cognition Composite	132.59 (14.23)	133.42 (14.95)

Fluid Cognition Composite	129.00 (13.72)	123.72 (14.13)
Dimensional Change Card Sort	107.17 (9.60)	107.52 (6.89)
Flanker	114.02 (4.36)	112.19 (3.71)
Picture Sequence Memory	113.53 (13.49)	110.46 (15.22)
List Sorting Working Memory	106.13 (14.68)	101.11 (9.89)
Pattern Comparison	120.93 (15.13)	115.00 (18.99)
Crystallized Cognition Composite	106.40 (9.40)	116.50 (16.33)
Picture Vocabulary Test	102.49 (9.13)	111.38 (14.81)
Oral Reading Recognition Test	109.73 (11.33)	114.80 (12.99)

Means and standard deviations in parentheses.

### Data Analytic Plan

Data analysis occurred in four steps^1^. First, global differences in Overall Cognition Composite Score were examined using a two group independent sample *t*-test. Second, differences in the Crystallized and Fluid Cognition Composite scores were examined using a multivariate analysis of variance (MANOVA). Third, an additional MANOVA compared groups for individual tasks within the fluid and crystallized cognition domains. Finally, to further illuminate the associations between cognition and social anxiety symptoms a series of exploratory, non-independent Pearson correlations were conducted.

## Results

### Are There Between Group Differences in Overall Cognition?

To examine whether the high and low socially anxious participants significantly differed in overall cognition, a two-group independent-sample *t*-test was conducted. Results indicated no between-group difference, *t*(47) = –0.200, *p* = 0.842.

### Are There Between Group Differences in Fluid or Crystallized Cognition?

Group differences in Fluid and Crystallized Cognition Composite scores were examined by a MANOVA. Cognitive performance was significantly different between the high and low anxious groups, *F*(2,46) = 6.023, *p* = 0.005, ${\text{η}}_{p}^{2}$ = 0.208. Follow-up pairwise analyses revealed that high socially anxious adults (*M* = 116.50, SE = 2.65) had significantly better performance than low anxious adults (*M* = 106.40, SE = 2.822) on the Crystallized Cognition Composite, *F*(1,47) = 6.790, *p* = 0.012, ${\text{η}}_{p}^{2}$ = 0.126. The high and low socially anxious groups did not differ in their fluid cognition performance, *F*(1,47) = 1.750, *p* = 0.192, ${\text{η}}_{p}^{2}$ = 0.036.

### Are There Between Group Differences in Individual Task Performance?

Additionally, performance on individual tasks within the fluid (Flanker, Dimensional Change Card Sort, Picture Sequence Memory, List Sorting, and Pattern Comparison) and crystallized (Picture Vocabulary and Oral Reading) cognition domains were examined using a MANOVA. Results indicated that there was a significant multivariate effect *F*(7,41) = 2.332, *p* = 0.042, ${\text{η}}_{p}^{2}$ = 0.285. *Post hoc* analyses revealed the high socially anxious adults (*M* = 111.38, SE = 2.45) performed significantly better than the low socially anxious adults (*M* = 102.49, SE = 2.60) on the Picture Vocabulary task *F*(1,47) = 6.196, *p* = 0.016, ${\text{η}}_{p}^{2}$ = 0.116, but did not differ in their performance on the Flanker (${\text{η}}_{p}^{2}$ = 0.051), Dimensional Change Card Sort (${\text{η}}_{p}^{2}$ < 0.001), Picture Sequence Memory (${\text{η}}_{p}^{2}$ = 0.012), List Sorting (${\text{η}}_{p}^{2}$ = 0.041), Oral Reading (${\text{η}}_{p}^{2}$ = 0.043), and Pattern Comparison tasks (${\text{η}}_{p}^{2}$ = 0.030).

### Are Deficits in Crystallized Cognition Associated with Severity of Social Anxiety Symptoms?

In order to further investigate the previously reported pairwise differences, exploratory, non-independent correlations were conducted between the LSAS total score and performance on all of the crystallized cognition measures. Results indicated that the LSAS was significantly and positively correlated with the Crystallized Cognition Composite score *r*(47) = 0.367, *p* = 0.009. Within the crystallized composite, LSAS total score was significantly correlated with the Picture Vocabulary task *r*(47) = 0.350, *p* = 0.014, but failed to reach significance with the Oral Reading task *r*(47) = 0.225, *p* = 0.120.

## Discussion

The present study used NIH Toolbox Cognitive Battery to compare cognitive abilities in young adults with high relative to low levels of social anxiety. Results indicated that high and low socially anxious participants did not differ in measures of fluid cognition. However, when compared to their low socially anxious peers, high socially anxious individuals showed advantages in crystallized cognition. Thus, the NIH Toolbox Cognition Battery may be a valuable standardized assessment for relating individual differences in cognition to individual differences in psychiatric symptoms.

In-line with attentional control theory (Eysenck et al., 2007), we hypothesized that we may see deficits in fluid cognition (specifically inhibitory and shifting processes) in participants with high social anxiety, however this pattern was not observed. While unexpected, there are a few reasons we may not see differences between groups. First, attentional control theory predicts that shifting and inhibitory deficits will be most pronounced when there is high load placed on the central executive. Given the developmental flexibility of the Toolbox, it is possible that Toolbox tasks do not place a high enough load on the central executive to observe group differences. Furthermore, attentional control theory allows that anxious individuals may be less efficient in their shifting and inhibitory processes, yet they may not show behavioral differences if anxious individuals evoke compensatory strategies (i.e., increased effort; Eysenck et al., 2007). Given that the NIH Toolbox does not measure such compensatory strategies, future studies should attempt to identify tasks that both put a high load on the central executive and assess compensatory strategy use (e.g., cognitive control or dual-task paradigms).

The lack of relations between social anxiety and fluid cognition was not entirely unexpected, given that null findings in this area are not uncommon (O’Toole and Pedersen, 2011). Additionally, to our knowledge, the Pattern Comparison, List Sorting Working Memory, and Picture Sequence tasks have not been used with socially anxious populations. The Dimensional Change Card Sort has been previously shown to moderate the relationship between behavioral inhibition and later anxiety symptoms in children (White et al., 2011), but other studies have failed to find an association between anxiety and performance on the closely-related Wisconsin Card Sort in adulthood (Graver and White, 2007). Furthermore, the lack of findings on the Flanker task were not entirely unexpected given many studies do not find behavioral differences between anxious and non-anxious adults (Moser et al., 2008; Dennis and Chen, 2009). Finally, given our moderate to small sample size, it is important to note that only medium to large effects could be detected (*f* = 0.33). Future studies should replicate the current results with larger samples to enable the detection of smaller effects.

The unreliability of past findings calls into question whether a third variable may mediate the relations between social anxiety and fluid cognition. One such variable may be state anxiety. There is some evidence for this claim since prior studies have found relations between fluid cognition and social anxiety when evocative stimuli (i.e., valenced faces and threatening words) are used (e.g., Mattia et al., 1993). Furthermore, studies using stress paradigms have shown decreases in performance on the Dimensional Change Card Sort (Graver and White, 2007) and changes in error monitoring on the Flanker (Barker et al., 2015). Given this pattern of results, future studies should investigate whether the negative relation between anxiety and fluid cognition only emerges when both state and trait anxiety are elevated.

Data from the current study also suggest that young adults with social anxiety show increased performance in crystallized cognition. To our knowledge, the current study is the first to examine crystallized cognition in a high socially anxious sample by using a multi-task standardized assessment battery. While both assessments administered have not been previously used with participants high in social anxiety, the current findings are in-line with a number of past studies suggesting socially anxious individuals may exhibit increased performance in scholastic achievement. For instance, Puklek Levpušček and Berce (2012) found that social anxiety was positively associated with grade point average in a sample of high-school students. One interpretation of these results is that socially anxious individuals spend more of their time in solitary activities (e.g., studying and reading), thus leading to gains in scholastic achievement.

However, other studies have not shown a similar relation between social anxiety and other measures of crystallized cognition on the Wechsler Adult Intelligence Scale (WAIS; Cohen et al., 1996; Graver and White, 2007). As an example, Graver and White (2007) found no difference between a small sample of participants with social phobia (*n* = 11) and healthy controls (*n* = 11) on the vocabulary subscale of the WAIS, which is similar to the Picture Vocabulary Test in the NIH Toolbox. A closer examination of the study by Graver and White (2007) revealed that the social phobic group outperformed the healthy controls by over a standard deviation, suggesting that with a larger sample, results may have been consistent with the present findings. Given the wide breadth of methods used in past research, the relation between social anxiety and crystallized cognition should be replicated using different samples with the same reliable and valid Toolbox measures presented here.

It is worth providing some cautionary notes to these results. First, while the high socially anxious group had LSAS scores near or above the clinical cutoff for anxiety (Mennin et al., 2002), current anxiety diagnosis and treatment information was not collected. Second, the current sample was comprised of students at a large state university, and therefore only reflects a small subset of all individuals impacted by social anxiety. Future studies should aim to replicate the current findings in larger, more diverse samples. Finally, the present sample was relatively homogenous in age and educational attainment. Future studies should investigate the NIH Toolbox using more heterogeneous samples in order to reveal whether controlling for demographic variables reveals different cognitive patterns in high and low socially anxious adults.

In sum, the current study advances our understanding of the relations between social anxiety and both fluid and crystallized cognition. Specifically, the data reveal that high socially anxious adults show no difference in fluid or general cognition but better crystallized cognition when compared to their low socially anxious peers. These findings suggest that there may be differential patterns of processing in the crystallized domain, which should be further investigated as a possible biomarker for anxiety. Finally, the NIH Toolbox Cognitive Battery is a valuable tool for assessing cognitive functioning in both normative and high socially anxious populations. In particular, the Toolbox’s comprehensive standardized scores, ease of administration, and the ability administer to participants 3–85 years of age, provides researchers with a powerful tool with which studies can be compared and replicated. In conclusion, these results suggest that social anxiety may influence crystallized cognitive abilities and suggests the use of the NIH Toolbox Cognitive Battery for future studies attempting to understand the relation between social anxiety and cognitive processes.

## Author Contributions

STR was the principal investigator of the study and the first author of the present manuscript. STR was involved with all parts of the present study including conception, data collection, data analysis, and manuscript preparation. STR is accountable for all aspects of the work. TB helped collect and analyze data, draft the present manuscript, and has approved the final version of the manuscript. TB is accountable for all aspects of the work. DP contributed substantially to the conception of the study and impacted data analysis. DP helped draft the present manuscript and approved the final version of the present manuscript. DP is accountable for all aspects of the work. NF made substantial contributions to all aspects of the present study. NF contributed to the conception, methods, data analysis, and manuscript preparation. NF has approved the final version of the present manuscript and is accountable for all aspects of the work.

### Conflict of Interest Statement

The authors declare that the research was conducted in the absence of any commercial or financial relationships that could be construed as a potential conflict of interest.

## Footnotes

1 Given that the NIH Toolbox Cognition Battery gives three separate composite scores for each participant (raw, age-adjusted, and fully-adjusted), analyses were completed for all three composites. All analyses yielded similar results. This was not unexpected due to homogeneity of the sample and high correlation between the three composite scores (*rs* > 0.5).
